# Supplementary material for: The impact of temperature on the transmissibility and virulence of COVID-19 in Tokyo, Japan
Source: Sci Rep. 2021 Dec 29;11:24477. doi: 10.1038/s41598-021-04242-3 (PMC8716537; doi:10.1038/s41598-021-04242-3)
Supplement: Supplementary file 1 — Supplementary Information. [file 41598_2021_4242_MOESM1_ESM.pdf]

The impact of temperature on the transmissibility and virulence of COVID-19 in Tokyo, Japan

Contents

1 Additional information on data collection **S2**

2 Description of daily temperature and daily number of cases and deaths **S3**

3 Time delay distribution from illness onset to death **S5**

4 MCMC diagnoses plot **S6**

5 Autocorrelation of R_t and CFR **S7**

6 Outcomes in the generalized additive models **S8**
6.1 Analysis of transmissibility S8
6.2 Analysis of severity S8

7 Sensitivity analysis **S10**
7.1 Distributed lag nonlinear model for R_t S10
7.2 Distributed lag nonlinear model for CFR S11

1 Additional information on data collection

We collected the daily number of confirmed cases, illness onset cases, deaths with COVID-19 in Tokyo, and the daily number of cases of emergency transportation whose destination had not been determined within 20 minutes from the start of the Emergency Medical Services team's request, or who had been refused by at least five medical institutions from Bureau of Social Welfare and Public Health, Tokyo Metropolitan Government. As for the human mobility data, we used Google's COVID-19 Community Mobility Reports. Daily weather data (mean temperature (℃), relative humidity (%), solar radiation as a ultraviolet (MJ/m^2), and mean wind speed (m/s)) were obtained from the Japan Meteorological Agency. Mean daily temperature (in ℃) and relative humidity (in %), were computed as the 24-hour average based on hourly measurements. Tokyo weather station located in Chiyoda-city was selected as representative.

2 Description of daily temperature and daily number of cases and deaths

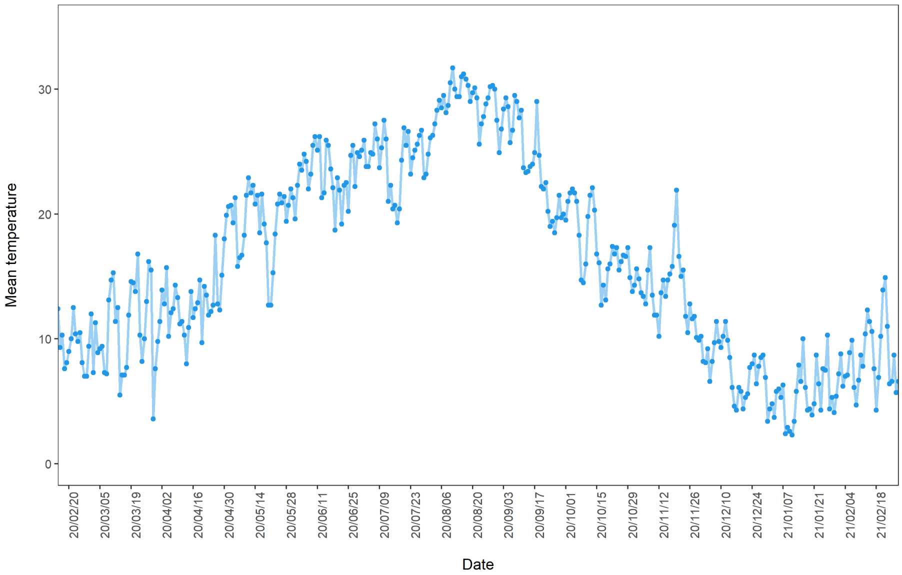

Figure S1: Daily mean temperature from 15th February 2020 to 28th February 2021 in Tokyo, Japan

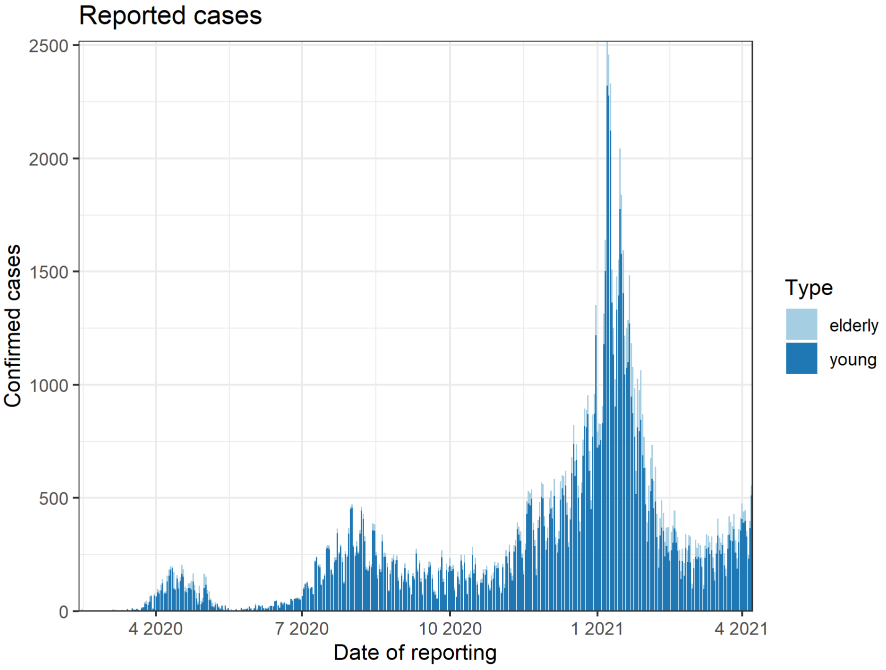

(a) Daily number of confirmed cases in Tokyo

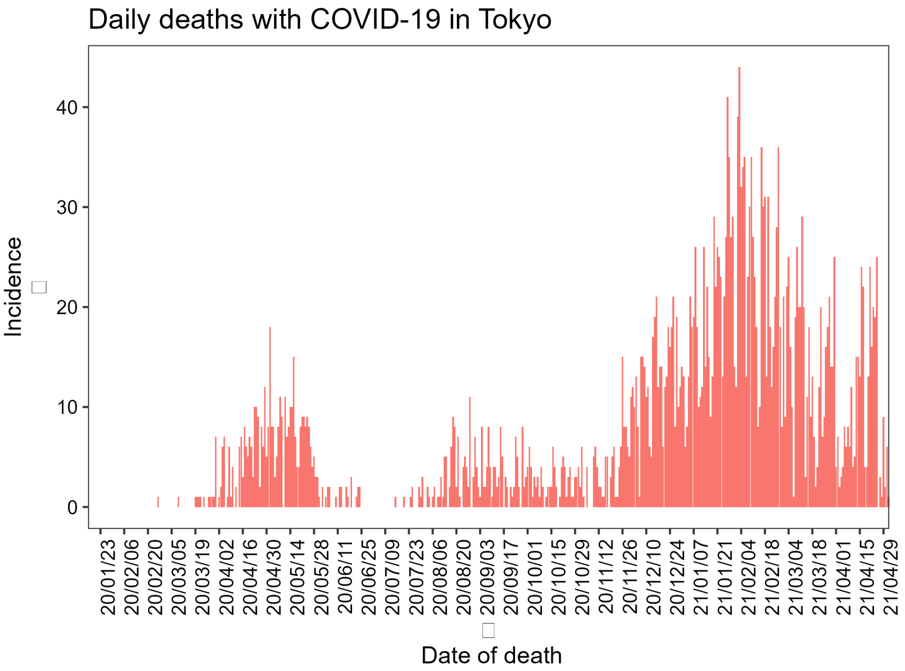

(b) Daily number of deaths in Tokyo

Figure S2: Daily number of cases and deaths with COVID-19

3 Time delay distribution from illness onset to death

We fitted empirical time delay distribution, which was collected from the publicly available data by the Tokyo Metropolitan Government, to Weibull, gamma, lognormal, and exponential distribution. Then gamma distribution was best fit. Figure S1 shows the empirical data and best fitted distribution. The mean and SD are 16.6 days and 118.4 days, respectively.

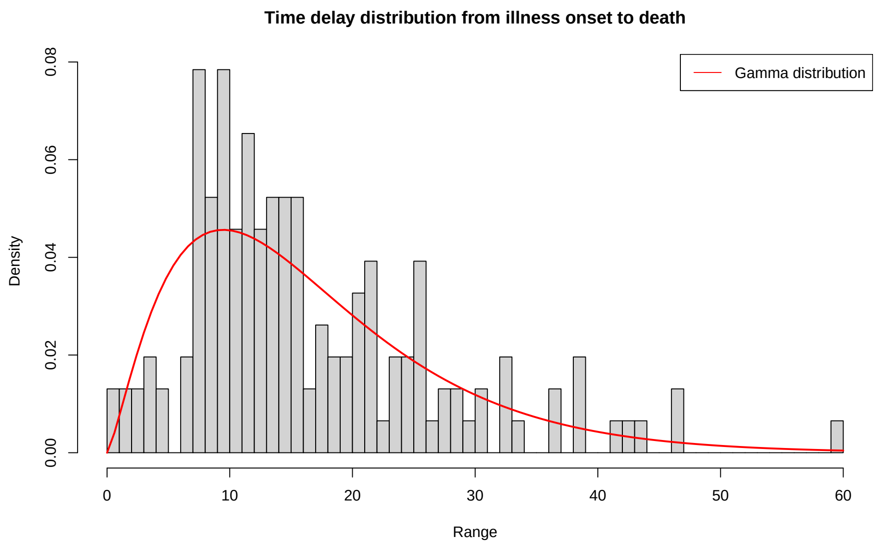

Figure S3: Empirical time delay distribution

4 MCMC diagnoses plot

In this section, the convergence of MCMC simulations for Baysian inference are shown in Figure S3. This results indicates that the unbiased CFR is estimated as 2.42% (95%CrI: 2.41-2.43).

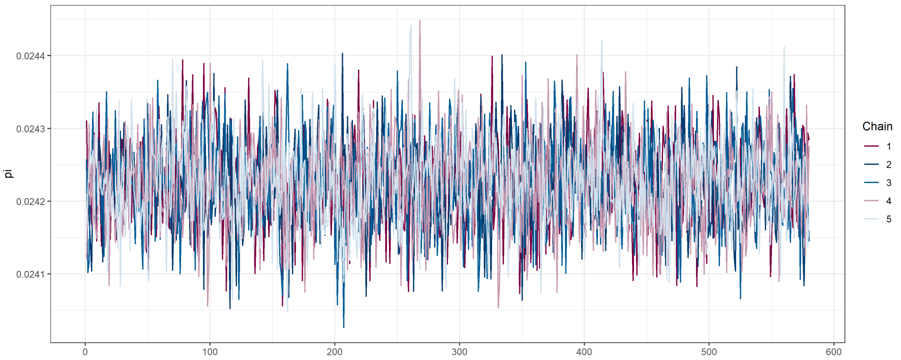

(a) Traceplot

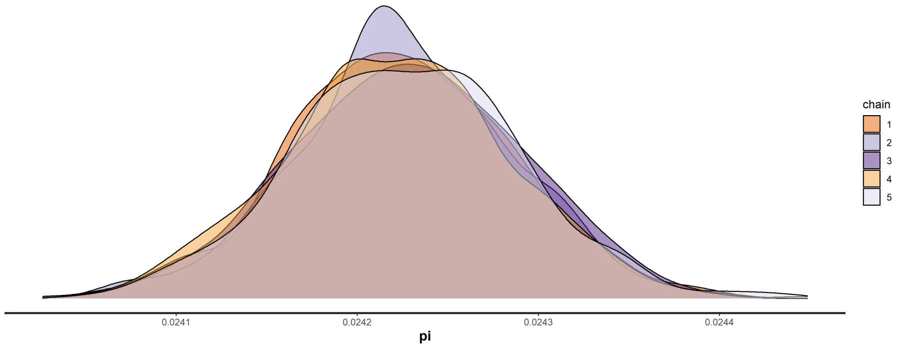

(b) Density plot

Figure S4: Adjustment for maximum lag up to 5 days

5 Autocorrelation of R_t and CFR

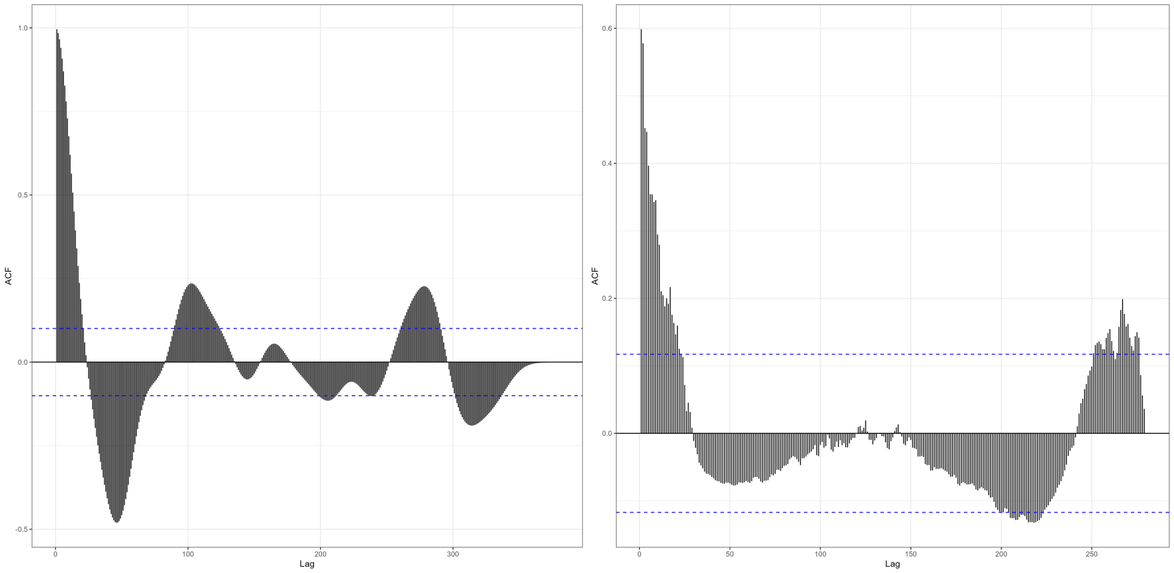

Figure S5: Correlogram of R_t and CFR

Left figure shows the autocorrelation of the estimated effective reproduction number and right figure shows that of time-delay adjusted temporal case fatality risk.

6 Outcomes in the generalized additive models

6.1 Analysis of transmissibility

Table S1 shows the RR of temperature by 2 days lag which is described in Figure 2. A&B.

Table S1: RR of R_t with stratified lags

Lag days	Extreme cold	Moderate cold	Moderate hot	Extreme hot
0-1	1.3 (1.1-1.5)	1.1 (1.1-1.2)	0.89 (0.81-0.98)	0.9 (0.8-1)
2-3	0.93 (0.88-1)	0.97 (0.93-1)	1 (0.96-1.1)	1 (0.96-1.1)
4-5	1 (0.98-1.1)	1 (1-1.1)	0.98 (0.94-1)	0.99 (0.94-1.1)
6-7	1.1 (1-1.1)	1 (0.99-1)	1 (0.97-1)	1 (0.98-1.1)

- **Extreme cold**: the first percentile of temperature (3.3 ℃), **Moderate cold**: the 10th percentile of temperature (6.3 ℃), **Moderate hot**: the 90th percentile of temperature (26.9 ℃), **Extreme hot**: the 99th percentile of temperature (30.6 ℃).

Table S2 shows the summary of generalized additive model (GAM).

Table S2: The summary of GAM for R_t

Variable	Estimate	SD	P-value
Intercept (α)	0.89	0.14	0.015
Intervention	-0.15	0.018	<0.0001
Residual	0.0063	0.0045	0.17
Workplace	0.003	0.0015	0.051
Retail and recreation	0.012	0.0013	<0.00001

6.2 Analysis of severity

Table S3 shows the RR of temperature by 3 days lag which is described in Figure 2. A&B.

Table S3: RR of CFR with stratified lags

Lag days	Extreme cold	Moderate cold	Moderate hot	Extreme hot
0-2	2 (1.2-3.5)	2.8 (1.7-4.7)	0.76 (0.53-1.1)	0.7 (0.44-1.1)
3-5	1 (0.85-1.3)	1.1 (0.9-1.3)	1.1 (0.97-1.3)	1.2 (0.98-1.4)
6-8	1.2 (1-1.3)	1.2 (1.1-1.3)	1.1 (1-1.2)	1.2 (1.1-1.3)
9-11	1 (0.91-1.3)	1.1 (0.92-1.2)	1.2 (1.1-1.3)	1.2 (1.1-1.4)
12-14	0.9 (0.8-1)	0.9 (0.84-1.1)	1.1 (1-1.2)	1.1 (1-1.2)

- **Extreme cold**: the first percentile of temperature (2.8 ℃), **Moderate cold**: the 10th percentile of temperature (5.8 ℃), **Moderate hot**: the 90th percentile of temperature (28.3 ℃), **Extreme hot**: the 99th percentile of temperature (30.8 ℃).

Table S4 shows the summary of GAM.

Table S4: The summary of GAM for CFR

Variable	Estimate	SD	P-value
Intercept (β)	-1.7	0.69	0.015
healthcare	0.0038	0.0026	0.15
age	-0.34	1.1	0.75
holiday	-0.18	0.14	0.21
weekMonday	0.12	0.1	0.23
weekSaturday	-0.13	0.097	0.19
weekSunday	0.15	0.1	0.15
weekThursday	0.18	0.098	0.07
weekTsuesday	0.1	0.095	0.29
weekTuesday	0.21	0.18	0.26
weekWednesday	0.098	0.097	0.31

7 Sensitivity analysis

We conducted sensitivity analysis to transmissibiity and severity by adjusting some possible meteorological confounders and the length of lags. As to the analysisof transmissibility, Figure S5 and S6 shows the results of changing the length of lags up to 5 and 6 days, respectively. Figure S7 shows the results of adjusting meteorological confounders. Here we considered relative humidity (%), solar radiation as ultraviolet (MJ/m^2), and wind speed (m/s).

As for the analysis of severity, we changed the maximum lag days up to 10 and 12 days (Figure S8 and S9). Relative humidity was also controlled in GAM (Figure S8). We chose a linear term for relative humidity because the least value of AIC indicates the 1st df for natural cubic spline was best fit for relative humidity (Figure S10).

7.1 Distributed lag nonlinear model for R_t

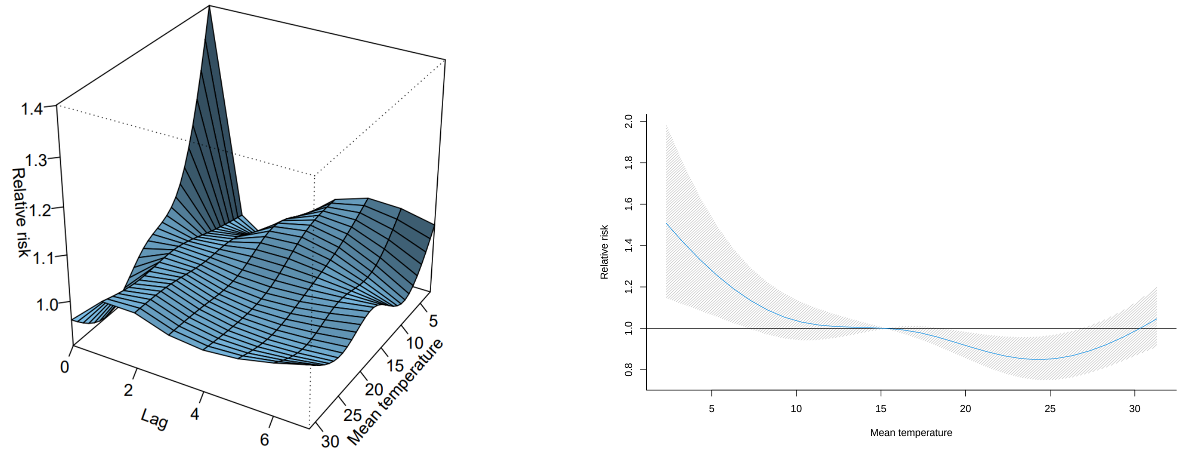

(a) Three-demensional plot

(b) Overall cumulative relationship

Figure S6: Adjustment for maximum lag up to 5 days

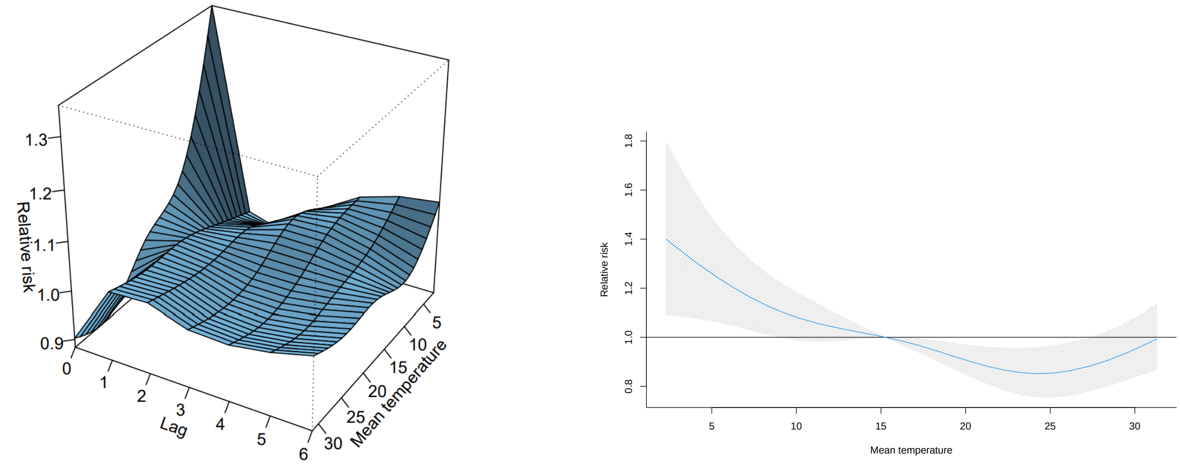

(a) Three-demensional plot

(b) Overall cumulative relationship

Figure S7: Adjustment for maximum lag up to 6 days

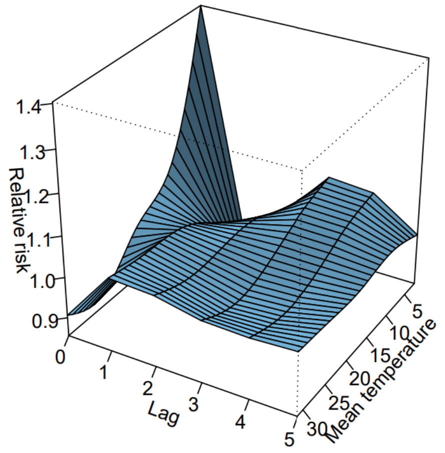

(a) Three-demensional plot

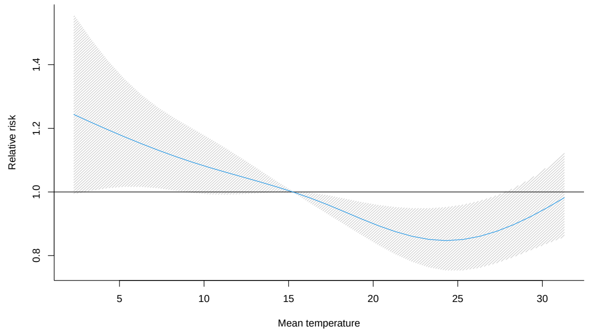

(b) Overall cumulative relationship

Figure S8: Adjustment for possible meteorological confounders

7.2 Distributed lag nonlinear model for CFR

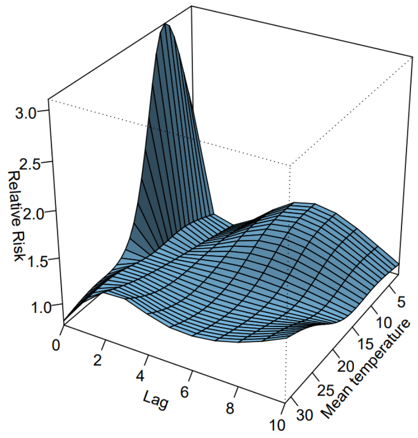

(a) Three-demensional plot

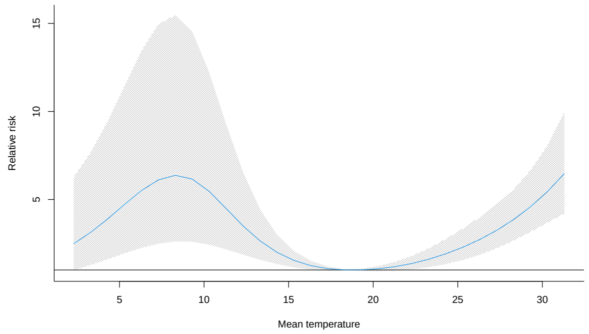

(b) Overall cumulative relationship

Figure S9: Adjustment for maximum lag up to 10 days

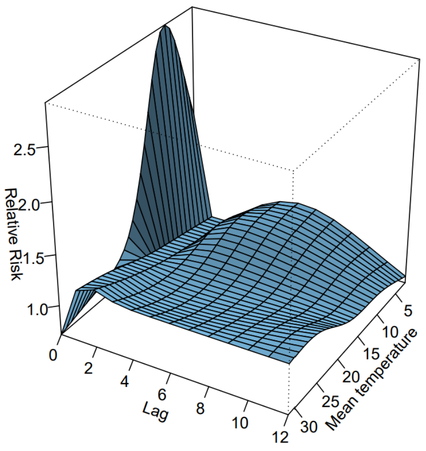

(a) Three-demensional plot

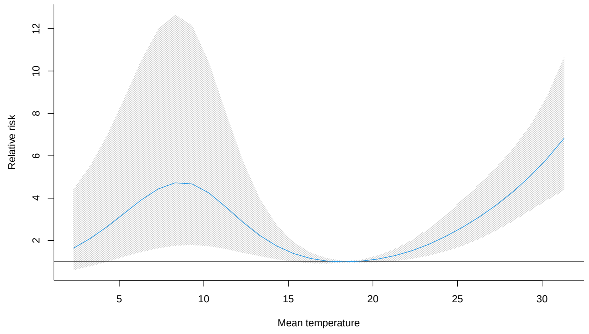

(b) Overall cumulative relationship

Figure S10: Adjustment for maximum lag up to 12 days

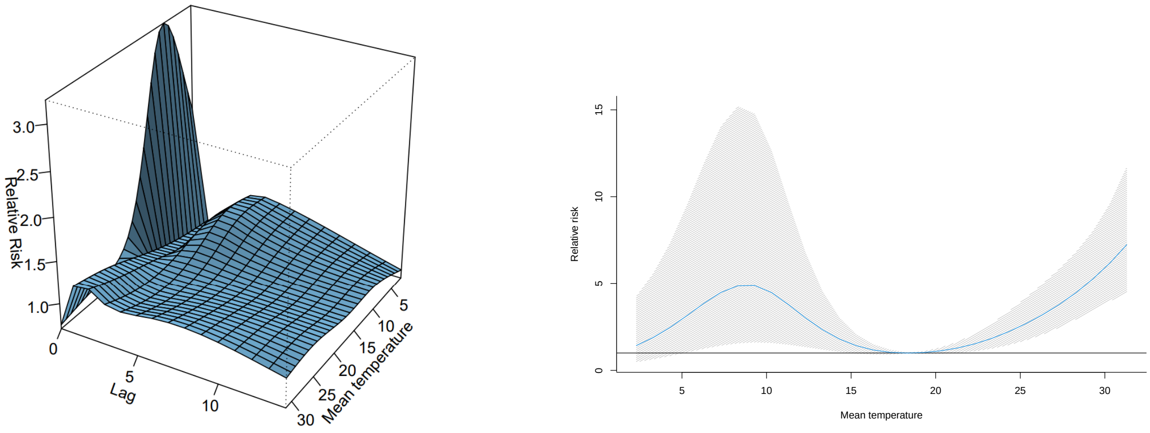

(a) Three-demensional plot

(b) Overall cumulative relationship

Figure S11: Adjustment for relative humidity
